# D1R- and D2R-Medium-Sized Spiny Neurons Diversity: Insights Into Striatal Vulnerability to Huntington’s Disease Mutation

**DOI:** 10.3389/fncel.2021.628010

**Published:** 2021-02-10

**Authors:** Guendalina Bergonzoni, Jessica Döring, Marta Biagioli

**Affiliations:** NeuroEpigenetics Laboratory, Department of Cellular, Computational and Integrative Biology (CIBIO), University of Trento, Trento, Italy

**Keywords:** Huntington’s disease, neurodegeneration, striatum, medium-sized spiny neurons, selective vulnerability, D1R, D2R

## Abstract

Huntington’s disease (HD) is a devastating neurodegenerative disorder caused by an aberrant expansion of the CAG tract within the exon 1 of the *HD* gene, *HTT*. HD progressively impairs motor and cognitive capabilities, leading to a total loss of autonomy and ultimate death. Currently, no cure or effective treatment is available to halt the disease. Although the *HTT* gene is ubiquitously expressed, the striatum appears to be the most susceptible district to the HD mutation with Medium-sized Spiny Neurons (MSNs) (D1R and D2R) representing 95% of the striatal neuronal population. Why are striatal MSNs so vulnerable to the HD mutation? Particularly, why do D1R- and D2R-MSNs display different susceptibility to HD? Here, we highlight significant differences between D1R- and D2R-MSNs subpopulations, such as morphology, electrophysiology, transcriptomic, functionality, and localization in the striatum. We discuss possible reasons for their selective degeneration in the context of HD. Our review suggests that a better understanding of cell type-specific gene expression dysregulation within the striatum might reveal new paths to therapeutic intervention or prevention to ameliorate HD patients’ life expectancy.

## Huntington’s Disease: Genetic, Clinic, and Pathologic Characteristics

Huntington’s disease (HD) is a rare, progressive, neurodegenerative disorder characterized by devastating motor, cognitive, and psychiatric symptoms. The monogenic, autosomal dominant disease is caused by a CAG repeat expansion in exon 1 of the HD gene (*HTT*), encoding for the huntingtin protein (MacDonald et al., 1993). The worldwide prevalence of HD is estimated to be 2.71 per 100,000 individuals (Pringsheim et al., 2012) and the average age of onset is between 30 and 50 years (Roos, 2010). So far, no treatments are available to block or slow-down the HD pathologic process, albeit mutant huntingtin lowering strategies are currently tested in clinical trials as promising therapeutic (Hoffmann-La Roche, 2020; Wave Life Sciences Ltd, 2020).

Although mutant huntingtin protein is ubiquitously expressed in all human districts, the brain, wherein the striatum, is the primary deteriorating region in HD (Saudou and Humbert, 2016; Ghosh and Tabrizi, 2018). Most striatal functions are mediated by inhibitory Medium-sized Spiny Neurons (MSNs), which comprise 95% of neurons in this area with the remaining being interneurons. There are two subtypes of MSNs differentiable by the expression of the D1 and D2 dopamine families’ receptors, constituting the direct and indirect pathways, respectively (Lanciego et al., 2012). The dorsal striatum (neostriatum) is the input module to the cortico-basal ganglia-thalamo-cortical loop (CBGTC), a neuronal circuit necessary for voluntary movement control. In the direct pathway, glutamatergic cortical terminals activate dopamine receptor 1 (D1R)-expressing MSNs, which exert their inhibitory effect on the globus pallidus internal segment (GPi) (*Entopeduncular nucleus*, in rodents). Inhibitory neurons in this area, project to the ventral anterior/lateral motor thalamus. Thus, the stimulation of D1R-MSNs has a net excitatory effect on the motor thalamus, allowing the final switch of the motor cortex and the stimulation of skeletal muscles. On the other hand, in the indirect pathway, dopamine receptor 2 (D2R)-expressing, inhibitory MSNs are also stimulated by glutamate release of cortical terminals. D2R neurons connect to the GP through an indirect loop, such that, they first project to and inhibit the globus pallidus external segment (GPe). These neurons firstly connect to the subthalamic nucleus exciting the area through glutamate release. Finally, the excitation of inhibitory GPi neurons produces motor thalamus repression (Alexander et al., 1986; Bolam et al., 2000). Dysfunction and death of striatal MSNs are the main causes for the motor disorders associated with HD (Ghosh and Tabrizi, 2018). In this review, we provide an overview of key pathological pathways leading to striatal degeneration. Furthermore, we describe general characteristics and physiological differences between D1R- and D2R-MSNs and highlight distinct morphological and functional alterations of MSNs during the disease. Our review emphasizes the importance of understanding cell-type specific physiological differences contributing to striatal vulnerability which may provide insights toward new avenues of therapeutic intervention.

## Pathogenic Mechanisms of the HD Mutation in Striatal Districts

### Altered Cellular and Molecular Pathways

**FIGURE 1:**
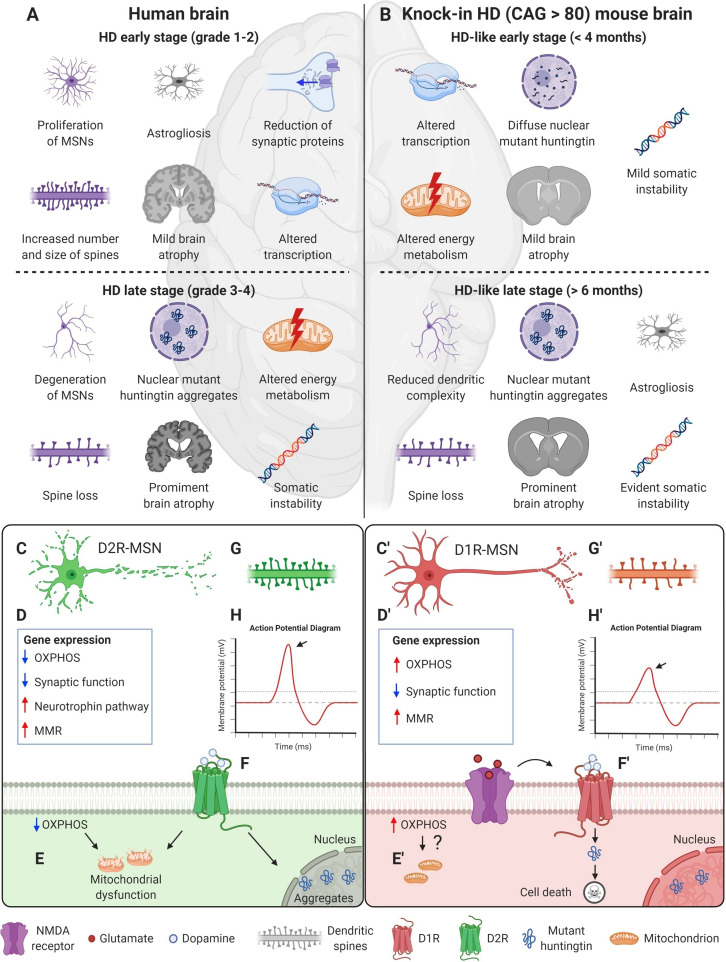
HD profoundly alters the striatum and MSNs in both patients and mouse models, upper panel. **(A)** Schematic representation of HD patient brain. At the early stage of the disease (grade 1–2), patients already manifest mild brain atrophy and astrogliosis (Ross and Tabrizi, 2011; Rüb et al., 2016). MSNs undergo proliferative expansion and show increased number and size of spines (Ferrante et al., 1991). At the molecular level, a reduction of synaptic proteins and alterations in gene transcription are detected (DiProspero et al., 2004; Hodges et al., 2006). These changes continue during advanced stages (grade 3–4), when brain atrophy becomes prominent (Rüb et al., 2016). MSNs undergo degenerative changes and spine loss, and mutant huntingtin aggregates can be detected within nuclei (Ferrante et al., 1991; DiFiglia et al., 1997; Rüb et al., 2016). Altered energy metabolism and somatic instability of the CAG tract are detected (Gines et al., 2003; Swami et al., 2009; Liot et al., 2017). **(B)** Knock-in HD mouse models (CAG > 80) faithfully recapitulate the human HD mutation and mimic several aspects of the human condition. At early age (<4 months), mice show mild brain atrophy (Peng et al., 2016) and diffuse accumulation of mutant huntingtin within MSNs nuclei (Carty et al., 2015). At the molecular level, energy metabolism and transcription are altered (Gines et al., 2003; Mochel et al., 2012; Ament et al., 2017). Somatic instability is already detectable at this stage (Pinto et al., 2013). At later stages (>6 months), brain atrophy becomes more prominent and astrogliosis could be detected (Menalled, 2005; Lerner et al., 2012; Peng et al., 2016). MSNs show reduced dendritic complexity and spine loss, and nuclear mutant huntingtin aggregates inclusions (Lerner et al., 2012; Carty et al., 2015). The molecular alterations proceed and somatic instability becomes particularly evident (Lee J.M. et al., 2011). Specific pathways and phenotypes developed by D1R- and D2R-MSNs upon expression of mutant huntingtin, lower panel. **(C,C′)** Striatopallidal neurons (D2R-MSNs) are affected earlier than striatonigral ones (D1R-MSNs) by HD mutation (Sapp et al., 1995). **(D,D′)** Gene expression profiling on R6/2 mice revealed that neurotrophin pathway is specifically upregulated in D2R-MSNs, while mismatch repair (MMR) and synaptic functioning pathways seem to be altered in both MSNs subpopulations (Lee et al., 2020). **(E)** In both R6/2 and zQ175 mice, oxidative phosphorylation (OXPHOS) downregulation observed in D2R cells contribute to mitochondrial dysfunction (Lee et al., 2020), while **(E′)** OXPHOS genes are upregulated in D1R cells, possibly suggesting a homeostatic response (Lee et al., 2020). **(F)** In mice and rat striatal cell cultures overexpressing mutant huntingtin, D2R stimulation enhances mutant huntingtin aggregation and mitochondrial dysfunction (Charvin et al., 2005; Benchoua et al., 2008). **(F′)** In *Hdh*^*Q111*^ models, dopamine and glutamate synergistically enhance MSNs sensitivity to mutant huntingtin toxicity through D1R activation (Paoletti et al., 2008). **(G,G′)** In 12 months old zQ175 KI mice, D1R neurons show proliferative expansion of the dendritic arborization and a significant reduction in the density of thin spines, while D2R neurons do not show significant differences (Goodliffe et al., 2018). **(H,H′)** In the same model, only D1R neurons exhibit reduced rheobase and action potential amplitude (arrows). Figure created with BioRender.com.

Because of unavailability of pre-symptomatic HD brain tissues, the reasons behind selective striatal vulnerability in HD were mostly investigated using animal models. In fact, the basal ganglia and, particularly, the cortico-striatal motor circuitry, appears to be conserved in mouse, minipig, and primates (Vodicka et al., 2005; Stephenson-Jones et al., 2011; Balsters et al., 2020). Thus, genetically engineered models, bearing normal or pathological CAG repeat lengths, have revealed important pathogenic mechanisms of the HD mutation (Menalled, 2005; Lerner et al., 2012; Peng et al., 2016; Table 1 and Figures 1A,B). Nevertheless, several salient features of human HD pathology–such as overt striatal atrophy, cortical degeneration, and onset of choreic movements–failed to fully replicate in animal models of the disease (Rubinsztein, 2002).

**TABLE 1:** Pathogenic mechanisms correlated with striatal degeneration in Huntington’s Disease.

Altered Mechanism*	Cellular and molecular phenotype*	References
BDNF-TrkB^1^ signaling^a^	Decreased BDNF synthesis and transport	Zuccato et al., 2003; Gauthier et al., 2004
Glutamate reuptake^b^	Glutamate excitotoxicity: decreased expression of, NMDA, AMPA, kainate, and excitatory amino acid transporter 2	Cha, 2007; Rebec, 2018
ROS^2^ production^b^	Increased: reduced expression of dopamine receptor D2R, nitric oxide synthase, and glutamate transporter GLT1	Cha, 2007; Kumar and Ratan, 2016
Mitochondrial functioning	Dysfunction: altered calcium homeostasis^c,d^, reduced ATP synthesize^e^, impaired mitochondrial trafficking^e^, mitochondrial fragmentation, and crestae alterations^d^ Dysregulation of electron transport chain genes^b,c^ and consequent alteration in OXPHOS^3^ complexes^b^	Panov et al., 2002; Seong et al., 2005; Costa et al., 2010; Li et al., 2010; Shirendeb et al., 2011; Liot et al., 2017
Gene expression^a,b,d,e,f^	Downregulated genes: neurotransmitter receptors, neurotransmitters, intracellular signaling molecules, and cytoskeletal/structural proteins Transcriptional changes also observed in glial cells	Luthi-Carter et al., 2000; Hodges et al., 2006; Cha, 2007; Tang et al., 2011; Ament et al., 2017
miRNA^4^ biogenesis and expression^d^	miRNA and miRNA biogenesis-related molecules are upregulated at earlier stages and downregulated at later stages of HD	Johnson et al., 2008; Packer et al., 2008; Lee S.T. et al., 2011
Alternative splicing	Aberrant: dysregulated *TRANS-*splicing factors (PTBP1, SRSF6)^b^. Mutant *HTT* mRNA sequesters spliceosome components, dysregulating splicing, and causing toxicity^g^	Sathasivam et al., 2013; Lin et al., 2016; Schilling et al., 2019
Epigenetics	Preferentially closed chromatin state and transcriptional repression: reduced histone acetylation, increased histone methylation^e,f^, decreased AcH3 levels, decreased number of genes bound by AcH3^f^, increased H3K27me3 and decreased H3K4me3^e^	Ferrante et al., 2003; Stack et al., 2007; Luthi-Carter et al., 2010; Seong et al., 2010; McFarland et al., 2012; Biagioli et al., 2015; Hervás-Corpión, 2018; Pearl et al., 2020
Dopamine signaling^b^	Altered dopamine signaling has been associated with behavioral alterations observed in HD. Dopamine levels are increased at early stage and decreased at later stage	Chen et al., 2013; Koch and Raymond, 2019
Somatic CAG instability^b^	Increased in striatum and cerebral cortex	Telenius et al., 1994; Swami et al., 2009
Electrophysiology^d^	Changes in the balance of excitatory and inhibitory inputs to the direct and indirect pathway MSNs	Galvan et al., 2012

*Abbreviations are indicated with superscript numbers, models used in the studies with superscript letters. ^1^Brain-derived neurotrophic factor-tropomyosin-related kinase receptor type B. ^2^Reactive oxygen species. ^3^Oxidative phosphorylation. ^4^MicroRNA. ^a^Knock-in mouse cell line (endogenous mouse *Htt* gene engineered to express a longer polyglutamine tract). ^b^HD patients. ^c^HD patients’ lymphoblastoid. ^d^Full-length mouse model (overexpression of full-length mutant huntingtin). ^e^Knock-in mouse model. ^f^R6/2 N-terminal mouse model (overexpression of human mutant *HTT* exon 1). ^g^Cell-line overexpressing mutant huntingtin.

Huntington’s disease *post-mortem* brains revealed that MSNs exhibit altered morphology, with proliferative changes–recurving and branching of dendrites and increased number and size of spines–since early stages of the disease. Degenerative alterations–truncation of the dendritic arborization and loss of spines–are characteristics of severe grades (Ferrante et al., 1991; Figure 1A). MSNs of 3 months old R6/2 N-terminal transgenic line–which overexpress human mutant *HTT* exon 1 (Mangiarini et al., 1996)- and of 20–26 months old *Hdh*^*Q140*^ knock-in mice–with the endogenous mouse *Htt* gene engineered to express a longer polyglutamine tract (Menalled et al., 2003)- do present similar decreased spine density and size of dendritic arborization (Klapstein et al., 2001; Lerner et al., 2012; Figure 1B).

Other studies in *post-mortem* brains also highlighted mutant huntingtin aggregates within neuronal MSNs nuclei (DiFiglia et al., 1997; Rüb et al., 2016; Figure 1A). Similarly, mutant huntingtin diffuse nuclear localization could be visualized at earlier ages (3 months) in MSNs of zQ175 knock-in mouse models (Menalled et al., 2012), while clear nuclear inclusions can be spotted only at later stages (8–12 months old) (Carty et al., 2015; Figure 1B).

Most observations point toward toxic gain-of-function for the pathogenic mechanisms. However, some data suggest that a loss-of-function mechanism should not be completely ruled out (Borrell-Pagès et al., 2006). Specifically, mutant huntingtin impairs the brain-derived neurotrophic factor-tropomyosin-related kinase receptor type B (BDNF-TrkB) signaling in striatal neurons (Table 1). This deficiency plays a pivotal role in dysfunction and death of MSNs and may represent a therapeutic target for HD treatment. Accordingly, several studies examined whether increasing levels of BDNF may be a viable strategy (Baydyuk and Xu, 2014). R6/2 mice, perfused with BDNF at 4 and 13 weeks of age showed less severe neurological dysfunction (Giampà et al., 2013), and significantly reduced motor coordination impairment (Giralt et al., 2011). However, the administration of citalopram, an antidepressant believed to increase BDNF levels, failed to improve motor and psychiatric symptoms in HD patients (Beglinger et al., 2014).

Recently, a significant increase in reactive oxygen species (ROS) production was described in the striatum of HD patients (Kumar and Ratan, 2016). ROS, produced by excitotoxicity or mitochondrial dysfunction, are important mediators of cell death (Gu et al., 1996; Browne et al., 1997). Coherently, mutant huntingtin seems to interfere with mitochondrial functioning (Table 1). Lymphoblasts from HD patients present mitochondrial fragmentation and cristae alterations (Costa et al., 2010), while cortical specimens from grade 3–4 HD patients display downregulation of complexes II, III, and IV of the oxidative phosphorylation (OXPHOS) pathway (Tabrizi et al., 2000; Shirendeb et al., 2011; Liot et al., 2017; Figure 1A). Energy metabolism alterations were also detected in R6/2 transgenic mice (Tabrizi et al., 2000), in *Hdh*^*Q111*^ knock-in models with decreased cAMP levels in the striatum at 10 weeks of age (Gines et al., 2003; Mochel et al., 2012) and, finally, precursor cells from striatal primordia of knock-in mice (Trettel et al., 2000) show significantly reduced respiration and ATP production (Figure 1B). Thus, considering that striatal neurons require higher amounts of ATP to maintain their hyperpolarized resting membrane potential (Hammond, 2015), it is conceivable that they might result more sensitive to mitochondrial dysfunction.

Initial studies on HD mouse models and later on HD *post-mortem* striatum revealed that mutant huntingtin causes transcriptional dysregulation of signaling pathways, neuronal, gliosis, and neuroinflammatory genes. Moreover, studies on *Hdh*^*Q111*^ knock-in models also demonstrated that transcriptional alterations can already be detected at 9 weeks of age (Cha, 2000; Luthi-Carter et al., 2000; Hodges et al., 2006; Ament et al., 2017; Table 1). Interestingly, striatal transcriptional changes are among the earliest detectable phenotypes in HD mouse models (Langfelder et al., 2016; Ament et al., 2017, 2018), which conform with HD patients (Seredenina and Luthi-Carter, 2012; Labadorf et al., 2015; Figures 1A,B).

Transcriptional dysregulation of synaptic proteins, such as complexin 2, dynamin, and PACSIN 1, correlates with neuronal morphological changes and reduction in the number of axonal fibers in early-stage HD patients (DiProspero et al., 2004; Han et al., 2010; Figure 1A). Furthermore, altered microRNA biogenesis and expression was reported in HD *post-mortem* tissues and in YAC128 murine models of full-length mutant huntingtin overexpression (Johnson et al., 2008; Packer et al., 2008; Lee S.T. et al., 2011; Table 1). Notably, mutant huntingtin can directly or indirectly compromise the epigenetic status of brain cells (Table 1), at least in part explaining the observed transcriptional dysregulation (Stack et al., 2007; Seong et al., 2010; McFarland et al., 2012; Biagioli et al., 2015; Hervás-Corpión, 2018; Pearl et al., 2020).

Recent RNAseq analysis of HD patients’ motor cortex revealed that mutant huntingtin interferes with RNA processing and induces aberrant alternative splicing (Table 1), affecting the expression levels of *TRANS-*splicing factors and/or trapping specific RNA binding proteins (Sathasivam et al., 2013; Lin et al., 2016; Schilling et al., 2019).

### Dopaminergic Signaling

Dopaminergic inputs from the *substantia nigra* are crucial for proper signaling of striatal MSNs in the basal ganglia circuit. Indeed, *substantia nigra pars compacta* (SNc) modulates the direct and indirect pathways by releasing dopamine, which has an excitatory effect on D1R and an inhibitory one on D2R. Consequently, dopamine excites the direct pathways and inhibits the indirect pathway, producing an overall stimulation of the motor activity (Leisman et al., 2013).

Studies on HD patients suggest that early stages of the disease are characterized by an increase in dopamine levels, contributing to choreiform symptoms. This might be due to the inhibitory effect of MSNs projecting to the SNc, which, in early stages, may produce hyperactivation of this pathway. Conversely, as disease progresses, dopamine levels decrease–possibly because of dopaminergic nigrostriatal terminals loss–accounting for the late akinetic stage (Chen et al., 2013; Koch and Raymond, 2019; Table 1). Accordingly, studies on both patients and mouse models confirmed an increase in dopamine release and tyrosine hydroxylase levels in early HD, followed by a reduction of the same parameters in advanced disease conditions (Koch and Raymond, 2019).

Within striatal MSNs, a modulatory mechanism between dopamine and glutamate was observed. On one hand, dopamine binding to D1R stimulates surface expression of NMDA and AMPA receptors, resulting in an increased responsiveness of D1R-MSNs to glutamate release. On the other hand, dopamine binding to D2R decreases surface AMPA receptors, reducing their glutamate excitability (Surmeier et al., 2007). Interestingly, both in patients and murine models, glutamate signaling follows the same pattern of dopamine alterations, being increased during HD early stages and decreased at advanced stages (Chen et al., 2013), thus suggesting a cross-talk between these two neurotransmitters.

### Somatic CAG Instability

The expanded CAG repeat in the mutant huntingtin gene is unstable, undergoing progressive length increases over time and resulting in somatic mosaicism in selective human body districts (Table 1). Specifically, it is possible that high level of somatic CAG instability in the striatum and cerebral cortex (Telenius et al., 1994; Swami et al., 2009; Lee J.M. et al., 2011) contributes to HD pathology (Figures 1A,B). Knock-out of DNA mismatch repair (MMR) proteins in *Hdh*^*Q111*^ knock-in mice showed that Msh2/3/6, Mlh1, and Mlh3 are modifiers of somatic CAG instability (Wheeler, 2003; Dragileva et al., 2009; Pinto et al., 2013). Importantly, genome-wide association analysis of a cohort of 9,000 HD patients confirmed MMR genes and specifically *MLH1* as crucial HD genetic modifiers (Lee et al., 2015, 2019).

## D1R- Versus D2R-Msns: General Characteristics and Physiological Differences

Medium-sized spiny neurons are characterized by a small to medium cellular body size (10–15 μm in diameter) and a radially oriented large dendritic tree covered by spines. Upon dopamine binding, D1R activates adenylyl cyclase (AC) signaling, leading to an excitatory effect, whereas D2R represses AC through Gi-protein signaling, resulting in inhibition (Lanciego et al., 2012). Striatopallidal (D2R) and striatonigral (D1R) neurons exhibit a random distribution in the murine rostral, dorsal striatum. However, a regionalization is observed in the caudal part, near the GPe, which comprises almost exclusively D1R-MSNs (Gangarossa et al., 2013). It is well accepted that D2R-MSNs are affected earlier than D1R-MSNs (Sapp et al., 1995; Figures 1C,C′) and, accordingly, GPe-targeting MSNs show substantial loss in patients at early stages of the disease (Albin et al., 1992). The lack of inhibition of the GPe by D2R-MSNs results in an excessive activation of the pallidal neurons, leading to choreiform movements observed in HD (Hedreen and Folstein, 1995). Nevertheless, in the latest stages, GPi-targeting MSNs of the direct pathway undergo marked decline, resulting in akinetic movements and rigidity (Deng et al., 2004; Lanciego et al., 2012).

Morphologically, striatonigral neurons show more primary dendrites and a more extended arborization than striatopallidal ones. Experimental simulation suggested that different dendritic areas may contribute to the divergent electrophysiological properties. Indeed, experiments performed on brain slices from D1R and D2R-EGFP BAC transgenic mice demonstrated that D1R neurons display a more hyperpolarized resting membrane potential and a greater rheobase (Gertler et al., 2008).

Accordingly, recent experiments using *Drd1a*-td Tomato mice revealed an increased intrinsic excitability for D2R-MSNs compared to D1R-MSNs. This might be due to the different rheobase, which is decreased in D2R neurons (Willett et al., 2019). Previous studies, however, pointed to a differential role of M1 muscarinic receptors activation, which downregulates Kir channel currents in striatopallidal MSNs, but not in striatonigral ones (Shen et al., 2007).

Considering that increased release of glutamate might contribute to MSNs degeneration (DiFiglia, 1990; Cepeda et al., 2007), it is noteworthy that D2R-MSNs receive more cortical inputs, mainly from pyramidal neurons (Francelle et al., 2014). Moreover, cortical axons making synapses with D2R-MSNs are larger in size, compared with the ones from D1R neurons (Lei, 2004). Altogether, these characteristics expose them to higher excitotoxicity, possibly reflecting on their greater susceptibility to cell death (Table 1). D2R-, but not D1R-, MSNs can form the protein complex with β-arrestin2, Akt, and protein phosphatase 2A (PP2A), which, in turn, reduces the phosphorylation of glycogen synthase kinase-3 (GSK3) (Harrison et al., 2013). GSK3 plays crucial roles in neuronal function, synapse formation, and neurite outgrowth (Beaulieu et al., 2004, 2005). Since both Rhes and Akt have been demonstrated to interact with and modulate mutant huntingtin toxicity, the Akt/β-Arrestin 2PP2A/GSK3 pathway may represent an additional mediator of D2R specific selective vulnerability (Colin et al., 2005; Lee et al., 2014).

Furthermore, TrkB is unequally expressed in striatal MSNs, with higher level in D2R-MSNs (Baydyuk and Xu, 2014). Thus, the aberrant BDNF-TrkB signaling caused by mutant huntingtin might have stronger effects in these cells (Table 1). Analysis of mouse striatum using single cell RNA sequencing (scRNA-seq) unveiled additional transcriptional differences between D1R- and D2R-MSNs. Further complexity emerged following the discovery of region-specific molecular markers for dorsal D2R neurons (Puighermanal et al., 2020), the identification of discrete subgroups of D1R and D2R neurons (Gokce et al., 2016) and of a possible third subtype of MSNs, which may have unique characteristics (Gokce et al., 2016). The existence of a third subpopulation of MSNs was also reported by Saunders et al. (2018), who observed a cluster of neurons in the striatum of C57BL6/N, co-expressing *Drd1* and *Adora2a*, named as “eccentric” MSNs. It is still premature to point to a clear connection between these physiological differences and the unequal cellular vulnerability to HD of the two MSNs subpopulations. Nevertheless, evaluation and integration of these single-cell analyses with other molecular aspects, such as alternative splicing, somatic mosaicism, and epigenetics differences between MSNs subtypes (Table 1), will be instrumental to understand the molecular mechanisms impinging on different vulnerability of D1R- and D2R-MSNs.

## Do D1R- and D2R-Msns Differentially Respond to the HD Mutation?

Recent studies on HD patients described rostro-caudal and dorso-ventral degenerative gradients. Specifically, the caudal striatum displayed greater neuronal death compared to the rostral part, while the dorsal-medial area seemed to degenerate faster compared to the ventral-lateral striatum (Morigaki and Goto, 2017). Although the contribution of MSNs’ physiological regionalization to selective vulnerability in HD is not fully dissected, some unequal distribution of the subclasses (Gangarossa et al., 2013) and subgroups (Gokce et al., 2016; Puighermanal et al., 2020) of MSNs might play a role.

To dissect why D1R- and D2R-MSNs are differentially affected by HD, Lee et al. (2020) highlighted thousands dysregulated protein-coding genes implicated in OXPHOS, synaptic functioning and circadian entrainment by using translating ribosome affinity purification and snRNA-seq of D1R and D2R neurons of HD patients and mouse models (R6/2 and zQ175DN, a knock-in zQ175 line without neomycin cassette) (Franich et al., 2019). Strikingly, downregulation of OXPHOS and upregulation of neurotrophin pathway genes in D2R neurons indicated a cell-type specific response to the disease (Figure 1D). Notably, Lee et al. (2020) demonstrated that OXPHOS genes downregulation causes mitochondrial dysfunction (Figure 1E) and mitochondrial RNA release in the cytosol, which, in turn, activates protein kinase R and cellular toxicity through the interferon pathways. Coherently, it was shown previously that D2R contributes to mutant huntingtin aggregation and mitochondrial impairment (Charvin et al., 2005, 2008; Benchoua et al., 2008; Figure 1F). Moreover, the upregulation of MMR genes, implicated in somatic instability of the CAG tract (Table 1), in both D1R- and D2R-MSNs supported a possible predisposing feature for selective degeneration (Figures 1D,D′). However, additional studies will be needed to correlate these findings with HD progression and MSNs vulnerability (Lee et al., 2020).

On the other hand, analysis on YAC128 and BACHD mouse models demonstrated that glutamate transmission was increased in D1R neurons at early disease and decreased in both D1R and D2R cells at advanced stages (André et al., 2011). Since a modulatory mechanism between dopamine and glutamate was observed within healthy striatal MSNs (Surmeier et al., 2007), dopamine and glutamate might synergistically enhance sensitivity to mutant huntingtin toxicity through D1R but not D2R activation (Paoletti et al., 2008; Figure 1F′). Similarly, in a different study using 12 months old zQ175 knock-in models, striatonigral neurons showed more prominent morphological and electrophysiological changes than striatopallidal ones (Goodliffe et al., 2018; Figures 1G′,H′). While this view is in contrast with the well-established hypothesis that D2R neurons are selectively damaged in early stages of HD, nonetheless, these findings might highlight the presence of a compensatory mechanism in D1R neurons. It is interesting to note that, in the knock-in mouse model zQ175DN, Lee et al. (2020) reported an increased expression for OXPHOS genes in D1R neurons (Figure 1D′), which, indeed, may support the activation of a transcriptional protective response in this subclass of MSNs.

## Conclusion and Perspective

In conclusion, our review provides a general overview into key pathological pathways leading to neuronal cell death of striatal MSNs in HD. We specifically focus on differences between D1R- and D2R-MSNs, underpinning sensitizing or protective features that might determine diverse responses to the same mutation. From initial studies, a combination of cell-type specific and non-specific reactions seem to be activated in HD, sensitizing D2R-MSNs to cell death. However, the application of single cell techniques, such as, but not limited to, scRNA-seq, is nowadays pioneering a new field of discussion, addressing the contribution of each single cell type (neuronal or glial) to HD striatal vulnerability. Specifically, other cell clusters in the striatum, such as striatal interneurons and astroglia, seem to respond to the HD mutation with some altered genes and pathways as in D1R and D2R, while microglia, oligodendrocytes, and oligodendrocytes precursors seem to be less responsive (Lee et al., 2020). The role of astrocytes in HD has been previously proposed, since mutant huntingtin downregulates the expression of the glial glutamine transporter GLT-1, exacerbating neuronal excitotoxicity. Similarly, specific mutant huntingtin expression in astrocytes prompts motor function deficits, weight loss, and age-dependent neurological phenotypes in transgenic mouse models (Bradford et al., 2009, 2010). Previous studies have provided evidence that activated microglia and reactive astrocytes might contribute to human HD pathology, perpetuating inflammation (Palpagama et al., 2019). However, still debatable is the attribution of beneficial vs. detrimental effects to activated microglia and astrocytes. Moreover, the highly heterogeneous class of striatal interneurons, generally thought to be spared in HD (Cicchetti et al., 2000), still displays selective degeneration in presence of the HD mutation, with documented loss of only parvalbumin-positive interneurons (Cicchetti et al., 2000; Reiner and Deng, 2018). Therefore, a better understanding of the neuroinflammatory environment, but also a detailed clarification of the interneurons population in the HD brain is needed. Moreover, analysis of chromatin, genome-architecture, and spatial distribution will assist in the elucidation of single cell characteristics. This will offer a new angle of interpretation to selective vulnerability to HD and will possibly pave the way to new avenues of therapeutic intervention.

## Author Contributions

GB made substantial contributions to conception, design of the work, and writing of the manuscript. JD assisted with drafting and critical reading of the text. MB conceived, supervised the project, and wrote the paper. All authors contributed to the article and approved the submitted version.

## Conflict of Interest

The authors declare that the research was conducted in the absence of any commercial or financial relationships that could be construed as a potential conflict of interest.
